# Antioxidant Defense, Redox Homeostasis, and Oxidative Damage in Children With Ataxia Telangiectasia and Nijmegen Breakage Syndrome

**DOI:** 10.3389/fimmu.2019.02322

**Published:** 2019-09-27

**Authors:** Mateusz Maciejczyk, Edyta Heropolitanska-Pliszka, Barbara Pietrucha, Jolanta Sawicka-Powierza, Ewa Bernatowska, Beata Wolska-Kusnierz, Małgorzata Pac, Halina Car, Anna Zalewska, Bozena Mikoluc

**Affiliations:** ^1^Department of Hygiene, Epidemiology and Ergonomics, Medical University of Bialystok, Bialystok, Poland; ^2^Clinical Immunology, The Children's Memorial Health Institute, Warsaw, Poland; ^3^Department of Family Medicine, Medical University of Bialystok, Bialystok, Poland; ^4^Department of Experimental Pharmacology, Medical University of Bialystok, Bialystok, Poland; ^5^Department of Conservative Dentistry, Medical University of Bialystok, Bialystok, Poland; ^6^Department of Pediatrics, Rheumatology, Immunology and Metabolic Bone Diseases, Medical University of Bialystok, Bialystok, Poland

**Keywords:** ataxia-telangiectasia (AT), nijmegen breakage syndrome (NBS), oxidative stress, oxidative damage, antioxidants

## Abstract

Ataxia-telangiectasia (AT) and Nijmegen breakage syndrome (NBS) belong to a group of primary immunodeficiency diseases (PI) characterized by premature aging, cerebral degeneration, immunoglobulin deficiency and higher cancer susceptibility. Despite the fact that oxidative stress has been demonstrated *in vitro* and in animal models of AT and NBS, the involvement of redox homeostasis disorders is still unclear in the *in vivo* phenotype of AT and NBS patients. Our study is the first to compare both enzymatic and non-enzymatic antioxidants as well as oxidative damage between AT and NBS subjects. Twenty two Caucasian children with AT and twelve patients with NBS were studied. Enzymatic and non-enzymatic antioxidants – glutathione peroxidase (GPx), catalase (CAT), superoxide dismutase-1 (SOD) and uric acid (UA); redox status—total antioxidant capacity (TAC) and ferric reducing ability of plasma (FRAP); and oxidative damage products−8-hydroxy-2′-deoxyguanosine (8-OHdG), advanced glycation end products (AGE), advanced oxidation protein products (AOPP), 4-hydroxynonenal (4-HNE) protein adducts, and 8-isoprostanes (8-isop) were evaluated in serum or plasma samples. We showed that CAT, SOD and UA were significantly increased, while TAC and FRAP levels were statistically lower in the plasma of AT patients compared to controls. In NBS patients, only CAT activity was significantly elevated, while TAC was significantly decreased as compared to healthy children. We also showed higher oxidative damage to DNA (↑8-OHdG), proteins (↑AGE, ↑AOPP), and lipids (↑4-HNE, ↑8-isop) in both AT and NBS patients. Interestingly, we did not demonstrate any significant differences in the antioxidant defense and oxidative damage between AT and NBS patients. However, in AT children, we showed a positive correlation between 8-OHdG and the α-fetoprotein level as well as a negative correlation between 8-OHdG and IgA. In NBS, AGE was positively correlated with IgM and negatively with the IgG level. Summarizing, we demonstrated an imbalance in cellular redox homeostasis and higher oxidative damage in AT and NBS patients. Despite an increase in the activity/concentration of some antioxidants, the total antioxidant capacity is overwhelmed in children with AT and NBS and predisposes them to more considerable oxidative damage. Oxidative stress may play a major role in AT and NBS phenotype.

## Introduction

Rare autosomal recessive disorders, Ataxia-telangiectasia (AT; OMIM #208900) and Nijmegen breakage syndrome (NBS; OMIM #251260) affect one in 40,000 to 100,000 people worldwide (1). They belong to a group of primary immunodeficiency diseases (PI) characterized by chromosomal breakage, chronic inflammation, premature aging, neurodegeneration as well as higher cancer susceptibility (2, 3). An elevated concentration of serum α-fetoprotein (AFP) is characteristic of children with AT whereas immunoglobulin deficiency and extreme radiosensitivity is typical of both AT and NBS patients (2, 3). On a molecular basis, AT and NBS result from mutations in the *Ataxia Telangiectasia Mutated (ATM)/NBN* genes that participate in signaling and repair of double-strand breaks in the DNA, cell-cycle control, apoptosis, chromosomal recombination as well as cellular senescence (3, 4). However, not all of the presented abnormalities can be explained by genomic instability, chromosomal rearrangements and immunodeficiency. It is speculated that oxidative stress also plays a key role in AT and NBS pathogenesis since a lack of ATM/NBS kinases disturbs signal transduction pathways responsible for redox (oxidative/reductive) homeostasis and free radical signaling (5–8). The mechanism by which ATM and NBS regulate redox status is not fully understood. Nevertheless, recent studies indicate that ATM promote glucose flux through the pentose phosphate pathway (PPP) in which the activity of glucose-6-phosphate dehydrogenase (G6PD) increases and NADPH production is generated needed for the regeneration of key antioxidant protein thioredoxin 1 (TRX1) (9, 10). Therefore, loss of ATM redox-sensing function may lead to the free radical accumulation in AT cells. Also in NBS patients, *NBN* mutations were inextricably linked with cellular redox imbalance due to the hyperactivation of Poly(ADP-ribose) polymerases, which leads to deficiency in DNA damage response pathway as well as ROS-induced DNA damage (11).

Oxidative stress is a harmful effect of reactive oxygen (ROS) and nitrogen (RNS) species, causing oxidative damage to cellular structures (12). It occurs when ROS/RNS are overproduced, endogenous antioxidant defense is impaired and the supply of exogenous free radical scavengers is reduced (12). Increased ROS production, mitochondrial dysfunction as well as more considerable oxidative damage have been shown in AT and NBS cell cultures (5, 7, 13). In AT cells, Yu et al. (14) demonstrated elevated ROS levels and decreased expression of antioxidant heme oxygenase (HO-1) associated with increased DNA fragmentation and free radical-induced apoptosis. Ambrose et al. (6) showed an aberrant structural organization of mitochondria in AT cells, a reduction in their membrane potential as well as increased expression of mitochondrial-targeted ROS detoxifying genes. It has also been demonstrated that in the animal model of NBS higher cancer incidence results from oxidative stress which is due to inadequate DNA damage response (11, 15). However, little is yet known regarding the role of *in vivo* redox abnormalities in the clinical manifestations of AT and NBS diseases (16). No studies assessing oxidative damage in patients with Nijmegen breakage syndrome have been conducted, either. Furthermore, the outcomes of human studies are frequently contradictory, which may be due to a small number of patients involved in the investigations. Therefore, the purpose of the study was to compare both enzymatic and non-enzymatic antioxidants, redox homeostasis and oxidative damage between AT and NBS children. The results of our investigation may contribute not only to a more thorough understanding of AT and NBS disregulation, but may also have considerable significance for clinical practice.

## Materials and Methods

The study was approved by the local Ethics Committee at the Medical University of Bialystok, Poland (permission number R-I-002/395/2014). Parents of all respondents gave written informed consent for their children's participation in the project.

### Patients

Twenty two Caucasian children with AT (mean age 13 years and 6 months, range 2-25 years, male/female 9/13) with a confirmed mutation in the *ATM* gene and 12 patients with NBS (mean age 15 years, range 4–26 years, male/female 5/7) with a confirmed mutation in the *NBN* gene were studied. The same mutation was identified in all patients with NBS (homozygous deletion (c.657_661del5) of *NBN* gene), whereas the mutations in AT patients are shown in Table 1. The diagnosis was established in accordance with the ESID (European Society for Immunodeficiencies) criteria. All participants were treated in the Department of Immunology, Children's Memorial Health Institute in Warsaw, Poland. Only patients in good general condition were included in the study. Full blood counts and biochemical profiles were within the normal limits and markers of inflammation were negative in all the participants prior to study commencement. The exclusion criterion was the presence of metabolic diseases (obesity, diabetes), heart disease, hypertension, liver, kidney and lung diseases, HIV infection, and cancer. The control group consisted of 34 healthy individuals matched for age and sex to AT and NBS patients. All subjects from the study and control groups had the same dietary habits and none was taking vitamins and antioxidant supplements. The clinical characteristics of patients with AT and NBS are presented in Table 2.

**Table 1:** Mutations in *ATM* gene in patients with AT.

**Patient**	***ATM*** **gene**	
	**allel 1**	**allel 2**
1	c.7630-2A>C	c.5932G>T
2	c.7630-2A>C	c.5932G>T
3	c.5932G>T	–
4	c.7630-2A>C	c.6145T>G
5	c.6095G>A	–
6	c.5932G>T	exon 62-63 deletion
7	c.1563_1564delAG	c.6145T>G
8	c.5549delT	c.7630-2A>C
9	c.6095G>A	c.8441delG
10	c.7630-2A>C	c.8287C>T
11	c.6095G>A	–
12	c.601C>T	c.748C>T
13	c.7630-2A>C	–
14	c.5932G>T	–
15	c.7630-2A>C	–
16	c.7630-2A>C	c.1563_1564delAG
17	c.5118_5121delAGAA	c.6095G>A
18	c.7630-2A>C	–
19	c.5932G>T	–
20	c.7630-2A>C	c.1563_1564delAG
21	IVS20-597_IVS20-582delAAGT	–
22	c.7630-2A>C	c.6095G>A

*AT, Ataxia Telangiectasia; ATM, Ataxia Telangiectasia Mutated*.

**Table 2:** Clinical characteristics of patients with AT and NBS.

		**AT**	**NBS**
Sex	Male *n*	9	5
	Female n	13	7
Age		15.00 ± 7.79	13.59 ± 7.86
BMI		17.10 ± 3.46	21.64 ± 5.78
Serum albumin (g/dL)		4.06 ± 0.21	4.500 ± 0.27
Total cholesterol (mg/dL)		185.30 ± 13.39	184.20 ± 6.89
HDL (mg/dL)		46.60 ± 4.39	75.33 ± 7.55*
LDL (mg/dL)		119.90 ± 17.08	125.50 ± 4.50
TG (mg/dL)		116.50 ± 16.86	110.20 ± 11.19
Glucose (mg/dL)		85.08 ± 3.05	85.22 ± 2.01
IgA (g/L)		0.47 ± 0.19	0.52 ± 0.07
IgM (g/L)		1.30 ± 0.14	0.85 ± 0.12
IgG (g/L)		9.09 ± 0.60	3.57 ± 0.52
ALC (cells/μL)		1595.00 ± 128.10	1592.00 ± 175.80
CD3 (cells/μL)		7.243 ± 0.712	5.453 ± 0.99
CD4 (cells/μL)		465.40 ± 53.57	517.60 ± 90.21
CD8 (cells/μL)		333.60 ± 42.83	286.00 ± 55.47
CD19 (cells/μL)		116.10 ± 22.2	158.30 ± 56.88
NK (cells/μL)		568.00 ± 59.78	392.20 ± 58.41
AFP (ng/mL)		202.30 ± 44.62	ND

*AT, Ataxia Telangiectasia; NBS, Nijmegen Breakage Syndrome; ND, no data*.

### Blood Samples

Following an overnight fast, venous blood (2 × 10 mL) was collected into tubes containing ethylenediaminetetraacetic acid (S-Monovette^®^ EDTA-K3, Sarstedt, Germany) and into tubes with a clot activator (S-Monovette^®^ Clotting Activator/Serum, Sarstedt, Germany). According to the manufacturer's instructions, blood samples were protected from light, centrifuged (2000 × g for 10 min at +4°C) and then plasma and serum were immediately separated. Blood was frozen at the temperature of −80°C until assayed.

### Redox Assays

The performed analysis included:

- Determination of enzymatic and non-enzymatic antioxidants—glutathione peroxidase (GPx, EC 1.11.1.9), catalase (CAT, EC 1.11.1.6), superoxide dismutase-1 (SOD, EC 1.15.1.1), and uric acid (UA);
- Determination of redox status—total antioxidant capacity (TAC) and ferric reducing ability of plasma (FRAP);
- Determination of oxidative damage products—advanced glycation end products (AGE), advanced oxidation protein products (AOPP), 4-hydroxynonenal (4-HNE) protein adducts, 8-isoprostanes (8-isop) and 8-hydroxy-2′-deoxyguanosine (8-OHdG).

Enzymatic antioxidants were analyzed in serum samples, while non-enzymatic antioxidants, redox status, and oxidative modification products in plasma samples. All determinations were performed in duplicate samples (except for CAT, SOD, and TAC; see below) and the final result was the arithmetic average of the two measurements. The results were standardized to 100 mg of the total protein. Total protein content was determined in duplicate samples using bicinchoninic acid assay (BCA) (17) with bovine serum albumin (BSA) as a standard (PIERCE BCA Protein Assay Kit, Rockford, USA). All reagents for biochemical assays (unless otherwise specified) were obtained from Sigma-Aldrich (Germany/United States). Absorbance/fluorescence was measured using the Infinite M200 PRO Multimode Microplate Reader (Tecan Group Ltd., Männedorf, Switzerland).

### Determination of Enzymatic and Non-enzymatic Antioxidants

GPx activity was measured using the method of Paglia et al. (18) based on the conversion of NADPH to NADP^+^. Absorbance was analyzed at 340 nm. One unit of GPx activity was defined as the amount of enzyme catalyzing the oxidation of 1 millimole of NADPH per min. CAT activity was determined spectrophotometrically by measuring hydrogen peroxide (H_2_O_2_) decomposition at 340 nm (19). One unit of CAT activity was defined as the amount of enzyme degrading 1 micromol of H_2_O_2_ per min. CAT activity was determined in triplicate samples. SOD activity was assayed spectrophotometrically by measuring the cytosolic activity of SOD by inhibiting the oxidation of epinephrine to adrenochrome (20). Absorbance was analyzed at 340 nm. It was assumed that one unit of SOD activity inhibited the oxidation of epinephrine by 50%. SOD activity was determined in triplicate samples. Plasma UA concentrations were estimated spectrophotometrically using a commercial kit (QuantiChrom™Uric Acid Assay Kit DIUA-250; BioAssay Systems, Harward, CA, USA) in accordance with the manufacturer's instructions. Absorbance was measured at 490 nm.

### Determination of Redox Status

Plasma TAC was analyzed in triplicate samples using 2.2-azinobis-3-ethylbenzothiazoline-6-sulfonic acid radical cation (ABTS^*+^) (21). Absorbance was measured at 660 nm. TAC levels were calculated from the calibration curve for 6-hydroxy-2,5,7,8-tetramethylchroman-2-carboxylic acid (Trolox). Plasma FRAP was determined spectrophotometrically by measuring the ferric reducing ability of samples with 2,4,6-tripyridyl-s-triazine (22). Absorbance was measured at 593 nm. FRAP levels were calculated from the calibration curve for FeSO_4_.

### Determination of Proteins, Lipids, and DNA Oxidation Products

Plasma AGE content was determined spectrofluorimetrically using the method of Kalousová et al. (23). The fluorescence of plasma samples was measured at the excitation wavelength of 440 nm and the emission wavelength of 350 nm. For AOPP determination, plasma samples were diluted 1:5 (*v:v*) in PBS (pH 7.2) (24). Plasma AOPP concentrations were analyzed spectrophotometrically by measuring the oxidative capacity of the iodine ion at 340 nm (23). For AOPP determination, plasma samples were diluted 1:5 (*v:v*) in PBS (pH 7.2) (25). Plasma 4-HNE, 8-isop and 8-OHdG concentrations were estimated using a commercial enzyme-linked immunosorbent assay (ELISA) in accordance with the manufacturer's instructions (Cell Biolabs, Inc. San Diego, CA, USA; Cayman Chemicals, Ann Arbor, MI, USA; USCN Life Science, Wuhan, China, respectively).

### Statistical Analysis

Data were processed using Statistical 12.0 (StatSoft, Cracow, Poland) and GraphPad Prism 7 (GraphPad Software, La Jolla, USA). The D'Agostino-Pearson and Shapiro-Wilk tests were used to examine the normality of result distribution. The results were expressed as mean ± SD. The Student's *t*-test was used to compare the study and control groups. In the absence of a normal distribution of results, the U Mann Whitney test was used. Relationships between examined parameters were determined by Spearman's correlation. Statistical significance was defined as *P* < 0.05.

## Results

### AT Patients

**Figure 1:**
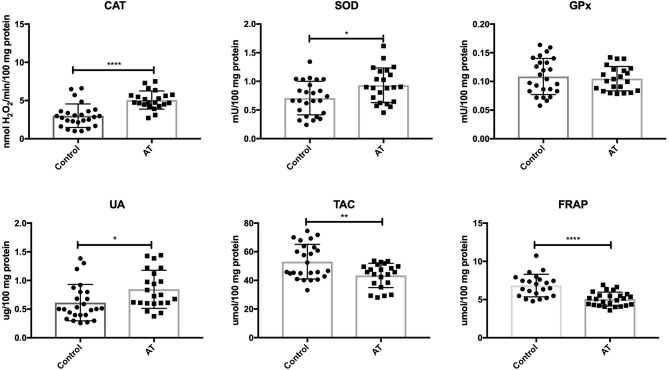
Enzymatic and non-enzymatic antioxidants in patients with AT and healthy controls. AT, Ataxia Telangiectasia, CAT, catalase; FRAP, ferric reducing ability of plasma; GPx, glutathione peroxidase; SOD, superoxide dismutase; TAC, total antioxidant capacity; UA, uric acid. ^*^P < 0.05, ^**^P < 0.005, ^****^P < 0.0001.

Our study demonstrated that the activity of CAT (*P* < 0.0001) and SOD (*P* = 0.0132) was significantly increased in AT patients in comparison with the control group. Out of the antioxidant enzymes, only GPx (*P* = 0.6401) did not differ statistically between the AT patients and healthy controls. UA concentration was significantly enhanced (*P* = 0.0185), while TAC (*P* = 0.0032) and FRAP (*P* < 0.0001) were statistically lower in the plasma of AT patients compared to controls (Figure 1).

**Figure 2:**
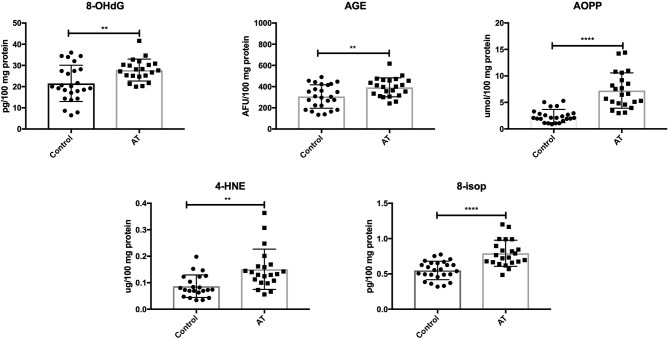
Oxidative damage in patients with AT and healthy controls. 4-HNE, 4-hydroxynoneal protein adducts; 8-isop, 8-isoprostanes; 8-OHdG, 8-hydroxy-2′-deoxyguanosine (8-OHdG); AGE, advanced glycation end products; AOPP, advanced oxidation protein products; AT, Ataxia Telangiectasia. ^**^P < 0.005, ^****^P < 0.0001.

We showed greater oxidative damage to DNA (↑8-OHdG; *P* = 0.0052), proteins (↑AGE, ↑AOPP; *P* = 0.0056, *P* < 0.0001) and lipids (↑4-HNE, ↑8-isop; *P* = 0.0014, *P* < 0.0001) in patients with AT compared to healthy children (Figure 2).

### NBS Patients

**Figure 3:**
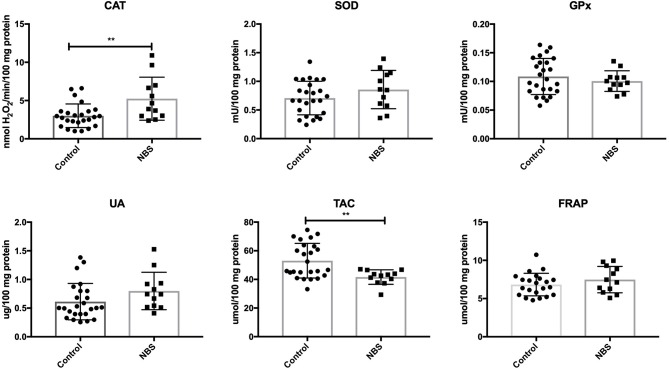
Enzymatic and non-enzymatic antioxidants in patients with NBS and healthy controls. CAT, catalase; FRAP, ferric reducing ability of plasma; GPx, glutathione peroxidase; NBS, Nijmegen Breakage Syndrome; SOD, superoxide dismutase; TAC, total antioxidant capacity; UA, uric acid. ^**^P < 0.005.

In patients with NBS, only CAT activity was significantly elevated (*P* = 0.0035), while the remaining enzymes (GPx and SOD) did not change significantly compared to the control group (*P* = 0.4231, *P* = 0.1763). UA levels did not change in patients with NBS vs. healthy controls (*P* = 0.107). Total antioxidant capacity (TAC) was significantly decreased (*P* = 0.0036), while FRAP was at the same level as in the control group (*P* = 0.2572) (Figure 3).

**Figure 4:**
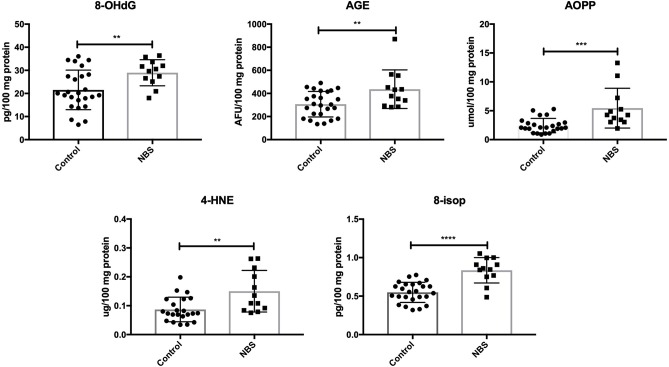
Oxidative damage in patients with NBS and healthy controls. 4-HNE, 4-hydroxynoneal protein adducts; 8-isop, 8-isoprostanes; 8-OHdG, 8-hydroxy-2′-deoxyguanosine (8-OHdG); AGE, advanced glycation end products; AOPP, advanced oxidation protein products; NBS, Nijmegen Breakage Syndrome. ^**^P < 0.005, ^***^P < 0.0005, ^****^P < 0.0001.

Similarly to subjects with AT, we showed higher concentrations of oxidative modification products: 8-OHdG (*P* = 0.0098), AGE (*P* = 0.0079), AOPP (*P* = 0.0005), 4-HNE (*P* = 0.0028), and 8-isop (*P* < 0.0001) in patients with NBS vs. the control group (Figure 4).

We did not demonstrate any significant differences in antioxidant defense and oxidative damage between patients with AT and NBS. Only the FRAP level was significantly higher in patients with AT compared to NBS (Table 3).

**Table 3:** Differences between antioxidant defense and oxidative damage in patients with Ataxia Telangiectasia and Nijmegen breakage syndrome.

	**AT**	**NBS**	***P***
**Enzymatic and non-enzymatic antioxidants**			
CAT (nmol H_2_O_2_/min/100 mg protein)	5.068 ± 0.253	5.244 ± 0.811	0.799
SOD (mU/100 mg protein)	0.932 ± 0.065	0.856 ± 0.096	0.505
GPx (mU/100 mg protein)	0.105 ± 0.005	0.101 ± 0.005	0.564
UA (μg/100 mg protein)	0.855 ± 0.071	0.780 ± 0.094	0.702
TAC (μmol/100 mg protein)	43.460 ± 1.807	41.650 ± 1.460	0.507
FRAP (μmol/100 mg protein)	5.062 ± 0.181	7.485 ± 0.498	<0.0001
**Oxidative damage products**			
8-OHdG (pg/100 mg protein)	27.810 ± 1.127	28.970 ± 1.629	0.552
AGE (AFU/100 mg protein)	393.100 ± 19.110	436.600 ± 48.220	0.328
AOPP (μmol/100 mg protein)	7.243 ± 0.712	5.453 ± 0.993	0.149
4-HNE (μg/100 mg protein)	0.151 ± 0.017	0.150 ± 0.021	0.986
8-isop (Pg/100 mg protein)	0.792 ± 0.039	0.853 ± 0.048	0.501

*4-HNE, 4-hydroxynoneal protein adducts; 8-isop, 8-isoprostanes; 8-OHdG, 8-hydroxy-2′-deoxyguanosine (8-OHdG); AGE, advanced glycation end products; AOPP, advanced oxidation protein products; CAT, catalase; FRAP, ferric reducing ability of plasma; GPx, glutathione peroxidase; NBS, Nijmegen Breakage Syndrome; SOD, superoxide dismutase; TAC, total antioxidant capacity; UA, uric acid*.

### Correlations

**Figure 5:**
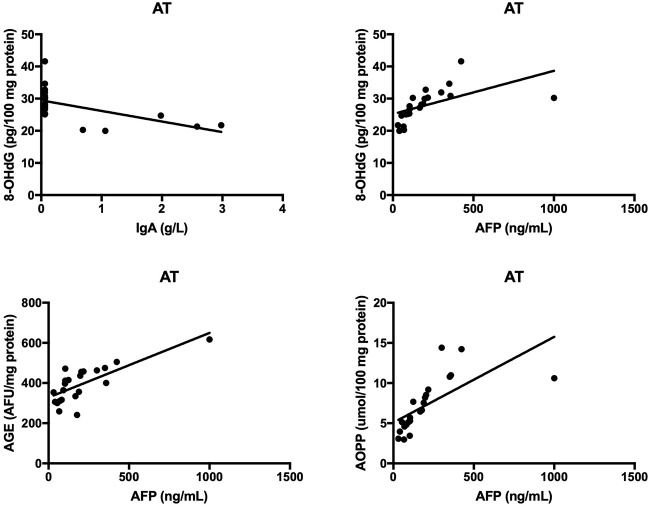
Correlations between oxidative stress biomarkers and clinical parameters in patients with Ataxia Telangiectasia. 8-OHdG, 8-hydroxy-2′-deoxyguanosine (8-OHdG); AGE, advanced glycation end products; AOPP, advanced oxidation protein products; AT, Ataxia Telangiectasia.

**Figure 6:**
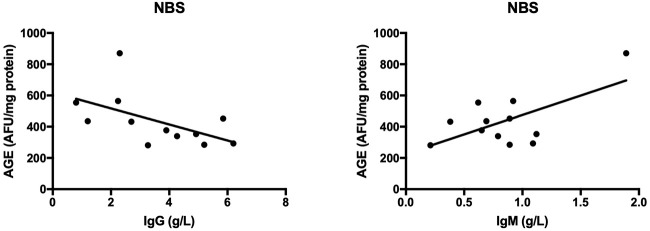
Correlations between oxidative stress biomarkers and clinical parameters in patients with Nijmegen breakage syndrome. AGE, advanced glycation end products; NBS, Nijmegen Breakage Syndrome.

The results of all statistically significant correlations are presented in Table 4. Importantly, we showed a positive correlation between the levels of 8-OHdG and α-fetoprotein as well as a negative correlation between 8-OHdG and IgA in AT. In AT children, AGE, and AOPP levels were also positively correlated with serum α-fetoprotein (Table 4, Figure 5). In NBS patients, AGE was positively correlated with IgM and negatively with the IgG level (Table 4, Figure 6).

**Table 4:** Correlations between redox biomarkers and clinical data of AT and NBS patients.

**Pair of variables**	***r***	***p***
**AT patients**		
CAT & BMI	0.390	0.050
AGE & AFP	0.750	<0.001
AGE & IgA	−0.465	0.029
AOPP & BMI	0.220	0.001
AOPP & AFP	0.669	0.001
AOPP & IgA	−0.516	0.014
AOPP & IgM	0.347	0.035
8-isop & CD19	0.444	0.044
8-OHdG & BMI	0.693	0.001
8-OHdG & AFP	0.556	0.007
8-OHdG & IgA	−0.572	0.005
**NBS patients**		
FRAP & IgG	−0.504	0.050
AGE & IgG	−0.547	0.050
AGE & IgM	0.631	0.038

*8-isop, 8-isoprostanes; 8-OHdG, 8-hydroxy-2′-deoxyguanosine (8-OHdG); AGE, advanced glycation end products; AT, Ataxia Telangiectasia; CAT, catalase; FRAP, ferric reducing ability of plasma; NBS, Nijmegen Breakage Syndrome*.

## Discussion

In our research, we evaluated selected biomarkers of redox homeostasis in 22 patients with AT and 12 patients with NBS. The present study is the first to compare both enzymatic and non-enzymatic antioxidants as well as oxidative damage between AT and NBS children. We demonstrated that AT and NBS are characterized by disturbances in blood redox equilibrium and enhanced oxidative damage to proteins, lipids, and DNA in comparison to healthy controls. However, we did not find significant differences between AT and NBS, which suggests a similar intensity of oxidative stress in these multisystem syndromes.

To date, the involvement of oxidative stress has been confirmed in the majority of currently known diseases including genetic conditions, neurodegenerative disorders, cardiovascular diseases, cancer, and aging (26–28). ROS are an inherent element of aerobic cell metabolism. Their overproduction, however, can result in damage to cell macromolecules and structures. The destructive activity of ROS includes lipid peroxidation, enzymatic proteins oxidation, protein aggregation/fragmentation as well as double-strand breaks in the DNA (29, 30). The essential role in preventing oxidative stress is played by enzymatic and non-enzymatic antioxidants. In this study, the elevated activity of blood antioxidant enzymes (↑CAT, ↑SOD) as well as an increase in plasma UA observed in AT and NBS patients suggest an adaptive body response to the overproduction of intracellular ROS (31). The high concentration of ROS in AT and NBS may be partially explained by a lack of differences in blood GPx activity. Both glutathione peroxidase and catalase participate in hydrogen peroxide (H_2_O_2_) decomposition. However, GPx operates at a low H_2_O_2_ concentration, while CAT at its high cellular level (32). It is well known that the induction of antioxidant enzymes is one of the most important mechanisms for limiting the production of ROS and regulating their biological activity (31). However, if chronic ROS overproduction occurs, the defensive antioxidant systems may be compromised or exhausted. Indeed, we showed that plasma antioxidant status is significantly lower in both AT (↓TAC, ↓FRAP) and NBS (↓TAC) patients. Considering that the total antioxidant potential is the resultant ability of all enzymatic and non-enzymatic antioxidants (21), a decrease in TAC and FRAP levels suggests disturbances in central redox homeostasis and a diminished ability to counteract oxidative stress. However, not only uric acid but also reduced glutathione (GSH) and total thiols (-SH) have a significant share in total antioxidant activity (21, 33). Although we did not assess GSH concentration in AT and NBS children, it is very likely that the decrease in plasma TAC and FRAP level may be caused by disturbances in glutathione metabolism. Indeed, GSH is one of the most important intracellular antioxidants, and its reduced synthesis and/or depletion of GSH reserves can be the main causes of redox abnormalities and associated oxidative stress (26). Therefore, the results of our research indicate the need to deepen the diagnosis of redox homeostasis in AT and NBS including assessment of reduced glutathione and other low molecular weight antioxidants.

In fact, redox imbalance in AT and NBS may result in cellular manifestations of the disease. The present study showed greater oxidative damage to proteins (↑AGE, ↑AOPP), lipids (↑4-HNE, ↑8-isop), and DNA (↑8-OHdG) in both AT and NBS children. Oxidative stress is closely related to neurodegeneration, immunodeficiency, cancer predisposition, and premature aging (34, 35), and thus accumulated oxidative damage may impact on some of the diverse symptoms of AT and NBS pathology.

Neurological abnormalities are one of the features shared by both AT and NBS (2, 3). As in neurodegenerative disorders, oxidative stress plays a critical role in the degeneration of the central nervous system (CNS) in AT and NBS patients (5, 36, 37). This is caused by the fact that the brain contains large quantities of polyunsaturated fatty acids (PUFAs) which are particularly vulnerable to oxidation by free radicals. Additionally, the brain consumes ~20% of oxygen supplied to the body, which, with intensive neuronal metabolism, results in the elevated production of ROS and RNS (26, 38). In this investigation, we showed greater oxidative damage to proteins (↑AGE, ↑AOPP) and lipids (↑4-HNE, ↑8-isop) in both AT and NBS patients. It appears that the oxidation of cellular proteins is the most important factor in neurodegeneration. Oxidatively modified proteins can form aggregates that are resistant to degradation by proteolytic enzymes. This promotes protein accumulation and leads to morphological changes in brain tissue (37). Additionally, it was shown that protein glycooxidation products (particularly AGE and AOPP) may also enhance ROS formation (e.g., by increasing NADPH oxidase activity), which further elevates the oxidative stress level (39, 40). AGE can also bind to a specific receptor (RAGE- receptor of advanced glycation end products) whose presence has been revealed on monocytes/macrophages, T lymphocytes, dendritic cells and neurons. In these conditions, AGE activates a number of transcription factors (e.g., NFkB, MAP-kinase, p21RAS) and adhesion molecules (e.g., ICAM and VCAM) leading to the formation of proinflammatory cytokines and chemokines (40, 41). Chronic inflammation is intensified, which, in addition to neurodegeneration, exacerbates immunity disorders or increases cancer predisposition (35). It is well known that lymphocytes are continuously active in AT and NBS. However, it is still unclear whether this is the cause or the consequence of the excess production of free radicals. Interestingly, we showed that AGE and AOPP levels were positively correlated with serum α-fetoprotein in AT patients, which indicates the participation of non-enzymatic glycation (carbonyl stress) in disease progression. α-fetoprotein increases with age and progression of AT and therefore, greater oxidative damage may be a pathogenic factor in the course of the disease. Similarly, the relationship between oxidative protein damage (AGE, AOPP) and immunoglobulin levels indicates the involvement of oxidative stress in the pathology of immunodeficiency.

Protein and lipid oxidation products may also react with nucleic acids, resulting in genetic mutations and chromosomal instability (42, 43). In the present study we demonstrated significantly higher levels of DNA oxidation marker 8-hydroxy-2′-deoxyguanosine in both AT and NBS children. Weyemi et al. (44) also showed increased 8-OHdG levels in AT patients, associated with the elevated expression of NADPH oxidase, enhanced ROS-induced apoptosis as well as progressive cerebellar degeneration (44). However, oxidative DNA damage is involved not only in neurodegeneration but also in cancer and premature aging (45). It is suggested that the extreme incidence of malignancies in AT and NBS may result from chronic ROS overproduction, deficiency in double-strand breaks repair and oxidative damage to nucleic acids. Despite the fact that most of the DNA oxidative damage is effectively removed by specialized DNA repair systems, these mechanisms are impaired in AT and NBS patients (4, 15). The DNA damage response pathway is a very complex signaling network in which many proteins are involved. The central function in the pathway is played by ATM and ATR (Ataxia telangiectasia Rad 3-related) kinases belonging to the PIKK (phosphatidylinositol 3-kinase-related kinase) family (42, 46). NBN protein also plays a similar role and therefore, the excess production of ROS in AT and NBS can cause reduced synthesis and/or activity of enzymes that remove DNA oxidative damage (15, 42). In addition, the increased formation of 8-hydroxy-2′-deoxyguanosine can modify genome methylation and DNA replication and, as a consequence, can change gene expression, induce carcinogenesis as well as accelerate telomere shortening since telomeric DNA (rich in guanine) is very sensitive to free radical action (36). Greater DNA oxidative damage may also be responsible for spontaneous apoptosis observed in lymphoblasts and lymphocytes of AT and NBS patients. Despite the fact that our study did not evaluate the molecular basis of AT and NBS, a positive correlation between the 8-OHdG and AFP levels as well as a negative correlation between 8-OHdG and IgA indicate a potential relationship between oxidative stress-related DNA damage and clinical manifestations of AT and NBS. Indeed, enhanced concentration of serum AFP is a reliable marker of AT after the age of 2 years (47). It was shown that ATM mutations are associated with inactivation of Arf/p53 tumor suppressor pathway resulting in higher AFP production but also in oxidative stress and ROS-induced ferroptosis (47–49). Similarly to hepatocytes, neurons are also vulnerable to oxidative DNA damage. AFP transports and reversibly binds PUFAs, among which docosahexaenoic acid (DHA) participates in brain maturation/myelinization and has a strong antioxidant properties. Therefore, it is proposed that oxidative stress can combine hepatic AFP generation, cerebral degeneration as well as higher cancer predisposition in AT children (47–49).

To date, only few studies evaluating oxidative stress biomarkers in AT and NBS patients (5, 7). Reichenbach et al. (50) observed a decrease in total antioxidant capacity/ plasma lipophilic antioxidants (vitamin A and E) in AT patients. They also reported enhanced level of total lipid peroxides and suggested that neurodegeneration in AT may be caused by higher oxidative damage do the membrane lipids. Degan et al. (51) indicated an adaptive response to oxidative stress evidenced by increase in blood oxidized glutathione (GSSG) and plasma methylglyoxal (MGlx) concentrations.

The results of our study demonstrate that redox abnormalities occurring in AT and NBS can impact on the clinical features of these disorders. However, it is very likely that AT and NBS have similar disturbances in oxidative/reductive status. Indeed, all assessed oxidative stress biomarkers (except FRAP) did not differ significantly between AT and NBS patients. Due to reduced antioxidant status, we indicate the usefulness of antioxidant supplementation in AT and NBS patients. Several studies have demonstrated that antioxidants (e.g., tempol, N-acetylcysteine, EUK-189, and CTMIO) can prolong survival in AT and NBS animal models (11, 52). Further studies are required to explore this issue in humans. It is worth noting that our previous study showed impaired antioxidant homeostasis in AT and NBS children which was primarily associated with deficiencies in plasma coenzyme Q10 and α-tocopherol (16). It is suggested that lipophilic antioxidants may support treatment and improve the clinical condition of AT and NBS patients (16). Therefore, there is a need for further studies to assess the clinical usefulness of antioxidants in AT and NBS patients and to evaluate the dynamics of change in relation to disease duration.

When analyzing the results of our research, one should also pay attention to its limitations. Firstly, we evaluated the most frequently used biomarkers of redox homeostasis and oxidative damage. Secondly, we demonstrated that the assessment of blood oxidative stress biomarkers has a peripheral value as it does not provide detailed information on redox homeostasis in organs particularly affected by AT and NBS. Since the main pathological processes in AT/NBS occur in the brain, we indicate the need for further research in an *in vivo* human model. However, the unquestionable advantage of our work is that a very large group of AT and NBS patients were investigated. This is also the first study which analyzed oxidative damage in NBS cases.

In conclusion, we demonstrated abnormalities in cellular redox homeostasis in AT and NBS patients. Despite an increase in the activity/concentration of some antioxidants, the total antioxidant capacity is overwhelmed in children with AT and NBS. We also showed that proteins, lipids, and DNA are indeed under oxidative stress and suffer oxidative damage in AT and NBS patients. However, the degree of redox imbalance is similar in these multisystem disorders. It is suggested that chronic oxidative stress may explain some of the diverse aspects of AT and NBS phenotypes. Therefore, more careful monitoring of ROS levels may be helpful in AT and NBS patients although the effectiveness of antioxidant supplementation remains an open question.

## Data Availability Statement

The datasets generated for this study are available on request to the corresponding author.

## Ethics Statement

The study was approved by the local Ethics Committee at the Medical University of Bialystok, Poland (permission number R-I-002/395/2014). Parents of all respondents gave written informed consent for their children's participation in the project.

## Author Contributions

MM: conceptualized, did laboratory determinations, performed statistical analysis, interpreted data, did performance of the graphic part of the manuscript, wrote the manuscript, reviewed the article, final approval of the version to be published. EH-P: conceptualized, performed clinical examination, qualified patients to the study, collected material, interpreted data. BP: performed clinical examination, qualified patients to the study, collected material. JS-P: collected material, interpreted data. EB: performed clinical examination, qualified patients to the study, collected material. BW-K: performed clinical examination, qualified patients to the study, collected material. MP: collected material. HC: interpreted data. AZ: conceptualized. BM: conceptualized, interpreted data, wrote the manuscript, reviewed the article, final approval of the version to be published, coordination of the project.

### Conflict of Interest

The authors declare that the research was conducted in the absence of any commercial or financial relationships that could be construed as a potential conflict of interest.

## Abbreviations

4-HNE: 4-hydroxynoneal protein adducts
8-isop: 8-isoprostanes
8-OHdG: 8-hydroxy-2′-deoxyguanosine (8-OHdG)
AFP: α-fetoprotein
AGE: advanced glycation end products
AOPP: advanced oxidation protein products
AT: Ataxia Telangiectasia
ATM: Ataxia Telangiectasia Mutated
ATR: Ataxia telangiectasia Rad 3-related
CAT: catalase
CNS: central nervous system
FRAP: ferric reducing ability of plasma
G6PD: glucose-6-phosphate dehydrogenase
GPx: glutathione peroxidase
GSH: reduced glutathione
GSSG: oxidized glutathione
HO-1: heme oxygenase-1
MGlx: methylglyoxal
NBS: Nijmegen Breakage Syndrome
PI: primary immunodeficiency diseases
PIKK: phosphatidylinositol 3-kinase-related kinase
PUFA: polyunsaturated fatty acids
RNS: reactive nitrogen species
ROS: reactive oxygen species
SOD: superoxide dismutase
TAC: total antioxidant capacity
TRX1: thioredoxin 1
UA: uric acid.
